# Antimicrobial Peptide Databases and Prediction Tools: Toward a Standard Evaluation Framework

**DOI:** 10.1021/acs.jcim.6c02141

**Published:** 2026-10-27

**Authors:** Alejandro Cisterna-García, Álvaro M. González-López, Aspasia Vozi, María A. Esteban, Adrian Egli, Catherine Jutzeler, Jose Palma, Álvaro Sánchez-Ferrer, Juan A. Botía

**Affiliations:** † Departamento de Ingeniería de la Información y las Comunicaciones, 16751 Universidad de Murcia, Murcia 30100, Spain; ‡ Biomedical Data Science Lab, Department of Health Sciences and Technology (D-HEST), 27219 ETH Zurich, Zurich 8092, Switzerland; § Institute of Medical Microbiology, University of Zurich, Zurich 8006, Switzerland; ∥ Departamento de Bioquímica y Biología Molecular-A, Facultad de Biología, Universidad de Murcia, Murcia 30100, Spain; ⊥ Department of Neurodegenerative Disease, UCL Institute of Neurology, London WC1E 6BT, U.K.; # Departamento de Biología Celular e Histología, Facultad de Biología, Universidad de Murcia, Murcia 30100, Spain; ∇ Department of Intensive Care and Neonatology, Children’s Research Center, University Children’s Hospital Zurich, University of Zurich, Zurich 8008, Switzerland; ○ SIB Swiss Institute of Bioinformatics, Lausanne 1015, Switzerland

**Keywords:** antimicrobial peptides, antibacterial peptides, antimicrobial resistance, machine learning, deep learning, AMP databases, AMP prediction, benchmarking, data leakage, reproducible evaluation

## Abstract

Antimicrobial resistance (AMR) has a profound impact on animal and human health and is associated with substantial morbidity, mortality, and public health costs. There is a clear need to develop novel, effective antibiotic agents, which can overcome the current AMR crisis. Antimicrobial peptides (AMPs) may offer such a solution and have attracted growing attention for their potential to combat AMR. In parallel, the growing availability of peptide sequences in public databases has stimulated the development of numerous machine learning and deep learning tools to predict antimicrobial activity computationally. However, it remains unclear how reliably these tools can be compared, as existing studies often rely on heterogeneous data sets and inconsistent evaluation protocols that may lead to data leakage and inflated performance estimates. This raises a central question: what evaluation criteria and benchmark resources are needed to enable fair, reproducible, and biologically meaningful assessment of AMP prediction tools? We address this question by focusing specifically on antibacterial peptides (ABPs). We first provide an overview of AMP databases relevant to antibacterial activity and compare their content, redundancy, and experimental metadata. We then critically assess existing computational tools for ABP prediction, highlighting key limitations related to data set construction, affinity to certain sequences, data leakage, and inconsistent performance reporting. Based on these limitations, we propose a reference evaluation framework designed to improve comparability, reproducibility, and practical utility in ABP prediction. Finally, we provide targeted recommendations for AMP databases and future tool development to support more robust progress in the computational discovery of ABPs.

## Graphical abstract

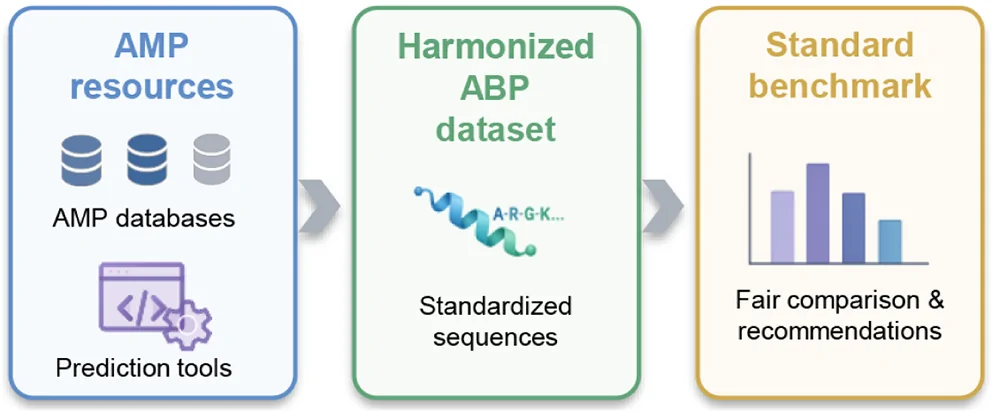

## Introduction

The number of deaths caused by complications derived from infections was around 13.7 million in 2019.^1^ Of these, 4.95 million were associated with pathogens carrying antimicrobial resistance (AMR), often referred to as *superbugs*.^2^ In response to this growing threat, the World Health Organization (WHO) included AMR in its list of urgent global health challenges, highlighting the critical need for new treatments.^3^ Furthermore, WHO also published a list of their bacterial priority pathogens.^4^ The WHO report states that more efforts and investments in novel antimicrobials are needed to address these most impactful pathogens, which include high-burden AMR bacteria such as *Enterobacterales*, particularly carbapenemase-producing *Enterobacterales* (CPE); nonfermenting Gram-negative bacteria, including *Pseudomonas aeruginosa* and *Acinetobacter baumannii*; *Salmonella* and *Shigella* *spp*; *Neisseria gonorrhoeae*; and *Staphylococcus aureus*. Among promising options, antimicrobial peptides (AMPs) emerge as a potential source of new treatments against these highly resistant bacteria.^5–8^

AMPs are short amino acid sequences that are generally considered to be less than 50 amino acids in length,^9^ and are found in a wide variety of organisms, ranging from single-celled prokaryotes to multicellular eukaryotes, including insects, amphibians, plants, and humans.^10–12^ AMPs have been routinely collected and classified in various databases, such as the Antimicrobial Peptide Database (APD),^13^ dbAMP,^14^ and the Antimicrobial Peptide Data Repository (DRAMP),^15^ based on their biological properties, including their antibacterial, antifungal, antiviral, and antiparasitic activities.^16–18^ The classical process of AMP discovery is complex, multistep, time-consuming, and resource-intensive. It usually begins with the identification of organisms, such as bacteria, fungi, plants, or animals, that produce bioactive peptides.^19–21^ Candidate peptides are then isolated from biological extracts or predicted from genomic, transcriptomic, or proteomic data, followed by purification and structural characterization using techniques such as chromatography and mass spectrometry.^22,23^ Once identified, these peptides must be synthesized or extracted in sufficient quantities and experimentally validated through in vitro assays against target pathogens.^24^ These assays conceptually assess pathogen growth in the presence of increasing concentration of the AMP. Promising candidates undergo further evaluation for stability, toxicity, selectivity, and mechanism of action, often requiring iterative rounds of optimization through sequence modification and structure–activity relationship studies.^25–27^ As a result, this traditional pipeline demands substantial experimental effort, specialized infrastructure, and long development timelines.

As an alternative to this tedious process, a series of computational tools have been developed that employ machine learning (ML) and deep learning (DL) approaches, leveraging the pattern-recognition capabilities of algorithms to identify patterns useful for distinguishing AMPs from non-AMPs.^28–53^ Typically, these predictive models are trained using annotated AMP sequences from AMP-specific databases and non-AMP sequences retrieved from general protein repositories, such as UniProt. The model’s performance is then typically evaluated using independent data sets of peptide sequences from other AMP databases. However, despite the proliferation of prediction tools based on ML and DL, the lack of a common data set across different tools makes direct comparison difficult and complicates the selection of the most appropriate tool.

Addressing existing research gaps in AMP discovery, this review focuses exclusively on antibacterial peptides (ABPs), prioritized for their clinical relevance and relative functional homogeneity. We introduce a novel benchmark that utilizes a comprehensive, integrated data set of ABP sequences compiled from the current database ecosystem (Figure 1). By systematically evaluating diverse AMP predictive tools against this unified and curated set, we draw attention to the critical limitations of existing models and propose a roadmap to refine predictive accuracy in this field.

**Figure 1:**
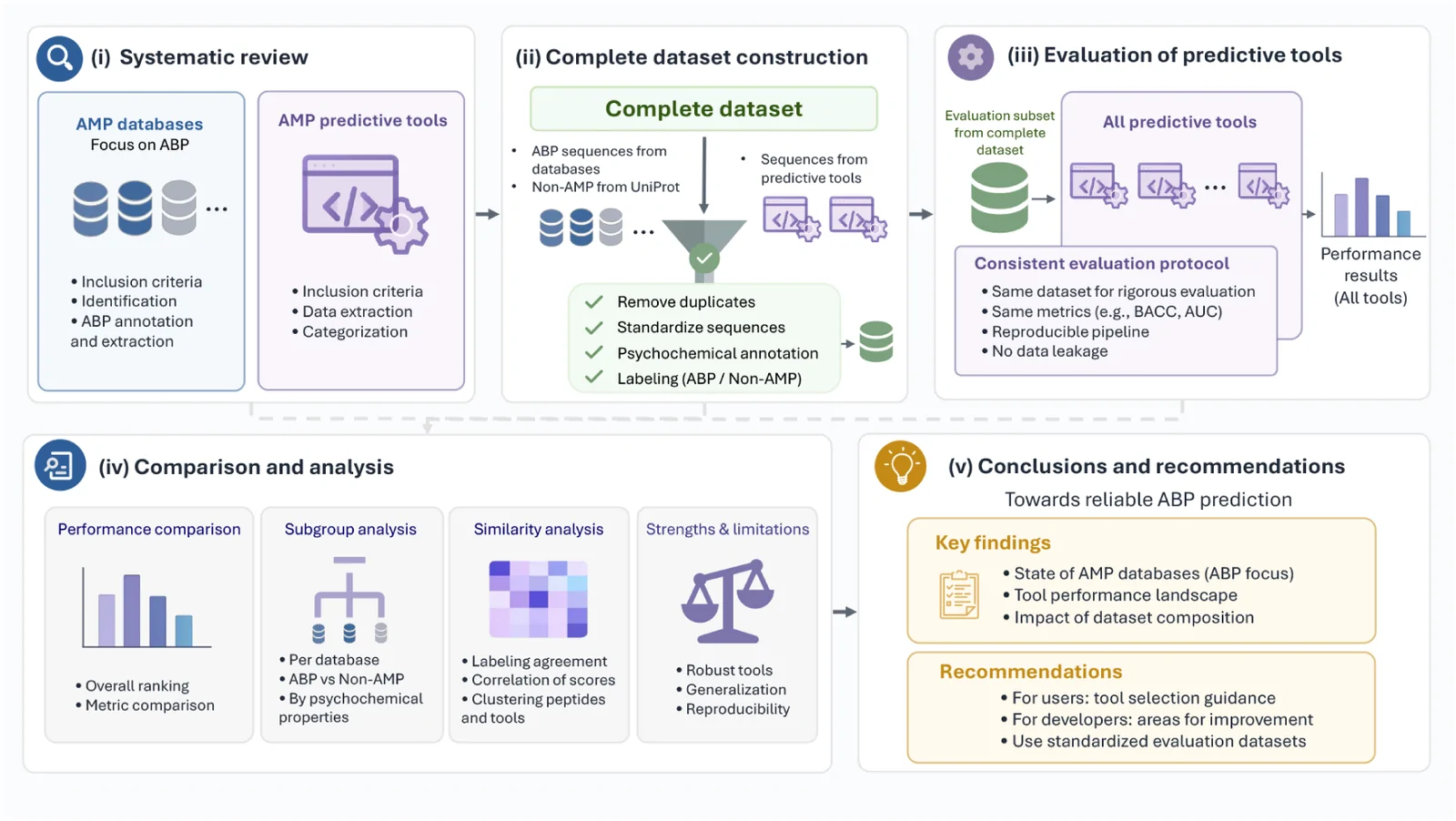
Overview of the AMP database review and benchmarking workflow. The following steps were taken into account to elaborate this review: (i) systematic identification of AMP databases and AMP predictive tools based on defined criteria; (ii) construction of a complete data set by integrating ABP sequences from curated databases, UniProt entries, and sequences retrieved from predictive tools, followed by duplicate removal, sequence standardization, psychochemical annotation, and labeling as ABP or non-AMP; (iii) evaluation of predictive tools using a consistent, reproducible protocol and standard performance metrics, including balanced accuracy, and AUC; (iv) subgroup analyses per database and tool to assess overall ranking, metric-level performance, and sources of bias, while identifying strengths and limitations related to robustness, generalizability, and reproducibility; and (v) synthesis of key findings into recommendations aimed at improving evaluation guidance, benchmarking practices, and the use of a common sequence data set for benchmarking AMP prediction.

## Overview of Antibacterial Peptides and AMP Databases

### Antibacterial Peptides: Mechanisms, Properties, and Challenges

The predominant class of ABPs consists of amphipathic, cationic α-helical structures. These peptides exploit electrostatic complementarity to selectively target the anionic components of microbial lipid bilayers, such as lipopolysaccharides (LPS) in Gram-negative bacteria or teichoic acids in Gram-positive bacteria. Upon sequestration at the membrane interface, the peptides undergo a conformational shift, leading to membrane permeabilization and subsequent osmotic lysis, in order to exert their bactericidal or bacteriostatic effects.^54–56^ However, not all ABPs are cationic, around 12% are classified as anionic or neutral peptides.^57^ This interaction with the bacterial membrane can result in its permeabilization through several distinct models, including the barrel-stave, carpet, and toroidal pore mechanisms.^58,59^ In all cases, the disruption of membrane integrity instigates a lethal cascade, encompassing dissipated ion gradients and the leakage of cytoplasmic contents, and ultimately cell death.^60^ Beyond these primary effects, ABPs frequently exert pleiotropic intracellular activities. Upon translocating across the lipid bilayer, these peptides have been observed to interfere with fundamental processes such as DNA replication, RNA transcription, protein synthesis, and enzymatic catalysis.^61,62^ This multitarget mechanism, which simultaneously compromises structural and metabolic components, presents a significantly higher evolutionary barrier to resistance compared to conventional single-target antibiotics.^10^ Beyond their direct antimicrobial actions, some ABPs exert significant immunomodulatory effects that bridge innate and adaptive immunity. A primary example is the human cathelicidin LL-37, which functions as a bifunctional effector by neutralizing pathogens while simultaneously recalibrating host immune signaling.^63^ These peptides orchestrate complex physiological processes, including leukocyte chemotaxis, the modulation of cytokine expression profiles, and the direction of cellular differentiation. Such multifaceted properties could be of particular therapeutic significance in the management of chronic wounds and persistent inflammatory pathologies where the resolution of immune dysregulation is a clinical priority.^64^

Despite their therapeutic potential, several physiological and economic constraints currently impede the widespread clinical translation of ABPs. A primary concern remains systemic cytotoxicity, as the biophysical mechanisms targeting bacterial membranes can inadvertently disrupt eukaryotic lipid bilayers, specifically manifesting as hemolysis in erythrocytes.^65–67^ To refine host selectivity, various structural stabilization strategies have been implemented, including peptide cyclization, the incorporation of D-amino acids, and hydrocarbon stapling.^68–70^ Furthermore, the clinical utility of ABPs is often limited by poor proteolytic stability in biological fluids, which severely curtails their systemic half-life and bioavailability.^65,71^ To address these pharmacokinetic deficiencies, advanced delivery platforms such as nanoparticle encapsulation, liposomal carriers, and hydrogel matrices are being deployed to shield peptides from enzymatic degradation and facilitate site-specific release.^72–75^ Finally, the high cost of large-scale solid-phase synthesis remains a significant barrier for complex or elongated sequences.^76,77^ Nonetheless, the advent of optimized recombinant expression systems and innovative bioengineering platforms has begun to address the challenges associated with scalability and production cost.^78–81^

Despite these hurdles, several ABPs have transitioned into clinical evaluation. However, regulatory approval by the Food and Drug Administration (FDA) and the European Medicines Agency (EMA) remains rare, with only a small number of candidates successfully navigating the clinical pipeline over the past 25 years.^82,83^ A notable success was daptomycin, a cyclic lipopeptide derived from *g* that received FDA approval in 2003 for the treatment of complicated skin and soft tissue infections. This agent remains a critical therapeutic for combating Gram-positive pathogens, including methicillin-resistant *Staphylococcus aureus* (MRSA) and vancomycin-resistant enterococci (VRE).^84,85^ On the other hand, colistin, also known as polymyxin E, is a last-resort antibiotic active against multiresistant Gram-negative strains of *Pseudomonas aeruginosa*, *Acinetobacter* *spp*, and *Klebsiella* *spp*, used both topically and parenterally, though the latter carries a risk of nephrotoxicity.^86^ More recently, the FDA-approved Dalbavancin in 2014, marking another milestone in peptide-based interventions.^87^ Current advancements in synthetic biology and peptide engineering are now facilitating the rational design of novel sequences with optimized antibacterial potency, stability, and pharmacokinetic profiles. These developments represent a transformative shift from the isolation of naturally derived molecules toward the creation of precision-engineered, next-generation peptide therapeutics.^88^

### Overview of AMP Databases

The primary sequences and physicochemical attributes of AMPs are curated within specialized repositories that facilitate the extraction of specific subsets based on biological origin or functional annotations. However, the inherent structural and functional heterogeneity across the AMP superfamily often hinders accurate classification and impedes direct benchmarking of predictive tools. Recognizing the clinical prominence of ABPs and the need for a common sequence data set for benchmarking ABP prediction, we retrieved and integrated all available ABP sequences from the databases summarized in Table 1 to assemble a comprehensive repository. This unified ensemble incorporates peptides acting against Gram-negative and Gram-positive bacteria to ensure an exhaustive overview of antibacterial activity, providing a more cohesive reference for evaluating current AI-based tools while establishing a refined training foundation for future model development.

**Table 1:** Summary of Sequence Retrieval Metadata across the Five Selected AMP Repositories^a^

Database	ABPs	Unique	Postprocessing	Nonstandard	Standard	Standard natural	Standard synthetic	Updated	Web
APD	5,496	5,494	5,494	15	5,479	3,073	2,024	2026	https://aps.unmc.edu/
DRAMP	4,159	4,049	4,010	91	3,919	2,665	1,383	2025	http://dramp.cpu-bioinfor.org/
dbAMP	7,625	7,622	7,619	756	6,863	–	2,553	2024	https://ycclab.cuhk.edu.cn/dbAMP/
DBAASP	20,980	17,970	17,009	2,550	14,459	2,699	11,487	2026	https://dbaasp.org/home
AMPDB	6,446	5,593	5,593	2,811	2,782	–	–	2023	https://bblserver.org.in/ampdb/

a For each database, the hosted ABP subset was retrieved, together with the natural and synthetic AMP subsets when available. All raw sequences underwent the same standardized preprocessing, including the removal of leading and trailing whitespace, conversion to uppercase, and the elimination of redundant entries. Nonstandard sequences were identified and labeled based on length constraints (outside the 5–255 amino acid range), the presence of nonalphabetic characters, or the inclusion of noncanonical residues. The ″Standard natural″ and ″Standard synthetic″ columns report the number of standard ABPs that also belong to the natural or synthetic AMP subset of the same database, respectively, after identical preprocessing. For APD, DRAMP, and DBAASP, the natural and synthetic subsets are those explicitly provided by each database; in contrast, for dbAMP, the synthetic subset was inferred from entries whose source annotation contains the terms ″synthetic″ or ″newly-designed″, and no natural subset could be defined. In these databases, the natural and synthetic categories may overlap, and they do not necessarily cover all database entries; consequently, their sum need not equal the number of standard sequences. An en dash (−) indicates that the database provides no such classification. The last update year reported for each database, as of the early-2026 eligibility assessment, is included. ABP subsets were downloaded on April 26, 2026, whereas natural and synthetic data were downloaded on September 15, 2026. APD, Antimicrobial Peptide Database; DRAMP, Data Repository of Antimicrobial Peptides; dbAMP, Database of Antimicrobial Peptides; DBAASP, Database of Antimicrobial Activity and Structure of Peptides; AMPDB, Antimicrobial Peptide Database.

Consequently, the present analysis focuses exclusively on the ABP subclass, applying the rigorous inclusion criteria summarized in Supplementary file 1 to select, as of early 2026, the databases that align with the scope of this review. This documentation enumerates both the repositories subjected to evaluation and those that were ultimately excluded for failing to satisfy the established selection standards. Specifically, 13 databases widely cited in the literature for their extensive curation of AMP data were evaluated. Of these, only 5 met the inclusion criteria, namely, the ability to filter for antibacterial peptides, evidence of maintenance or updates within the past three years, and public accessibility, and were therefore used to compile our data sets.

To ensure compatibility with the predictive architectures reviewed herein, which exclusively accept the 20 canonical amino acids represented in uppercase format, all retrieved entries underwent standardized preprocessing. This pipeline involved the removal of leading and trailing whitespace characters, conversion to uppercase, and the elimination of redundant sequences. The resulting peptide counts at each stage of this curation process are reported in Table 1, together with the composition of the standardized sets in terms of natural and synthetic peptides for each database, where available. Comprehensive documentation of the data acquisition process, along with source code used to download and process the data sets, is hosted on GitHub (https://github.com/Maximo-7/amp_review).

Established in 2003, the **APD** is one of the earliest AMP databases and was an influential resource for later AMP databases. It has evolved over 20 years into a versatile database that includes peptides up to 200 residues in length. It is the first database that sets up criteria for peptide registration.^13,89,90^ The database distinguishes between natural, synthetic, and predicted AMPs, provided they exhibit experimentally confirmed antimicrobial functions.^13^ The first three versions of the APD systematically collected peptide activity, post-translational modifications, 3D structures, and physicochemical descriptors, classifying peptides in multiple manners, including by source, activity, structure, covalent bonding patterns, synthesis machinery, and mechanism of action. The newest three versions have deployed and arranged a comprehensive information pipeline covering peptide discovery, in vitro data, and in vivo animal data. In vitro data include, for instance, peptide stability, hemolysis, synergy, bacterial resistance development, and mechanism of action, while in vivo data cover pharmacokinetics, toxicity, and efficacy in various animal models. The APD also contains information for peptide recombinant production, formulation, and clinical trials. All the information can be consulted and filtered via the APD search engine. Additionally, the platform integrates computational tools for multiple sequence alignment and AMP prediction. However, a notable technical constraint is that only sequence-ID pairs can be downloaded in bulk, with the richer metadata accessible solely through individual entry pages. Future improvements should focus on enabling users to download APD metadata in a structured format suitable for downstream AI-based prediction and modeling.

**DRAMP** serves as a comprehensive, curated open-access repository that categorizes AMPs into four distinct subsets: general, patent, clinical, and specific.^15,91^ The patent-derived data provide deep architectural insights through associated patent identifiers and abstracts, while the clinical category archives the progress of peptides currently in the therapeutic pipeline. Within the specific subset, DRAMP organizes specialized sequences into subgroups such as stapled and stability-enhanced peptides. A significant advantage of the DRAMP architecture is the systematic annotation of hemolytic activity and metabolic stability, which are indispensable for the rational design of clinical anti-infectives.^15^ These data sets, in conjunction with other sequence-specific metadata, are available in tabular formats. Furthermore, the platform offers functional integration with fundamental bioinformatics workflows, including sequence alignment and similarity-based searches.

**dbAMP** is a versatile repository that has supported the development of numerous predictive architectures since its inception in 2018.^14,92^ A distinguishing feature of dbAMP is its integration of structural biology with sequence data. It incorporates predicted tertiary structures for the majority of entries alongside over 100 docked complexes of AMPs and their respective target proteins. These structural models were generated utilizing ESMFold_V1 (Evolutionary Scale Modeling Fold) to provide high-fidelity insights into peptide-target interactions.^93^ The database architecture permits multidimensional filtering based on validation status, biological function, molecular target, toxicity, and structural annotations. Its functional subsets are highly granular, subdividing antibacterial peptides into specialized categories such as antilisterial and antibiofilm agents, among others. However, the utility of the database for large-scale training is hampered by restrictive download capabilities. Currently, the platform only provides a single file with limited metadata, requiring researchers to manually extract detailed annotations for specific peptide subsets. Beyond data hosting, dbAMP also provides integrated computational modules for predicting peptide half-life, hemolytic activity, and antibacterial efficacy.

**DBAASP** represents a foundational resource for structure–activity relationship (SAR) modeling and the *de novo* design of antimicrobial peptides.^94^ Its curated data sets have been instrumental in the development of numerous computational models and derivative databases since 2014.^95–106^ Unlike platforms that provide binary activity classifications, DBAASP prioritizes quantitative, experimentally determined susceptibility metrics. This includes exhaustive metadata regarding secondary structure, post-translational modifications, and covalent bond architectures, all contextualized within the specific experimental conditions of the assays. A distinctive technical advantage of DBAASP is its programmatic accessibility. Concise summaries are available for manual download, and the entire database is retrievable via a dedicated REST API^107^ that enables multidimensional filtering across all annotation fields. Furthermore, the platform integrates modules for physicochemical property calculation and species-specific activity prediction. Notably, DBAASP allows for the comparative ranking of entries based on quantitative potency against defined microbial strains or cell lines, a feature that distinguishes it from most extant AMP databases.

**AMPDB** is a recently established, large-scale repository that consolidates peptide data into 88 functional categories based on reported biological activities.^108^ While it is the largest database in this analysis by total entry count, its ABP subset is comparable in size to those of its peers. The platform serves as a meta-repository, aggregating and refining data from the NCBI Protein and EMBL-EBI databases with additional insights from primary literature. Each peptide record is extensively annotated with family classifications, molecular properties, and specific antimicrobial or enzymatic inhibitory functions, often linked via cross-references to external biological databases. A significant technical advantage of AMPDB is its data accessibility, offering structured tabular downloads for both the entire repository and its specific activity-based subsets. Beyond data hosting, the platform facilitates robust sequence analysis through the integration of multiple alignment tools (BLASTp [Basic Local Alignment Search Tool protein],^109^ MUSCLE [Multiple Sequence Comparison by Log-Expectation],^110^ Needleman-Wunsch,^111^ and Smith-Waterman^112^ and physicochemical calculators, making it a versatile resource for the preliminary stages of peptide discovery.

## Comparative Analysis of Antibacterial Sequences in AMP Databases

For the purpose of this review, we constructed a comprehensive repository by merging data from three primary sources: (i) ABP entries curated from the five aforementioned databases, (ii) non-AMP sequences retrieved from UniProt (criteria to select sequences from UniProt are defined in Supplementary file 1), and (iii) the training data sets associated with the predictive architectures evaluated in the subsequent sections. This integrated resource, hereafter designated as the *complete data set*, contains exhaustive metadata for each sequence. This includes cross-database identifiers, descriptive nomenclature, and Boolean attributes for subset classification, such as the designation of standard sequences or evaluation cohorts. Furthermore, the repository archives intrinsic physicochemical descriptors alongside the ground-truth labels and the class assignments generated by each predictive tool. These features are incorporated sequentially; physicochemical properties and model predictions are computed following the assembly of the primary data set, with tool performance evaluation derived from the final annotated ensemble. The distribution of physicochemical descriptors is of particular significance for comparative database analysis, as detailed in the following sections.

The subsequent analyses focus exclusively on standard sequences, defined as those composed of the 20 canonical amino acids with lengths restricted to a range of 5 to 255 residues. These parameters were established to align with the input constraints of the various AMP prediction tools, ensuring broad compatibility while minimizing data attrition. Regarding the ABP cohort, we observed varying sequence counts across the repositories with significant degrees of overlap. The distribution of unique sequences contributed by each database, alongside all possible combinations, is illustrated in Figure 2.

**Figure 2:**
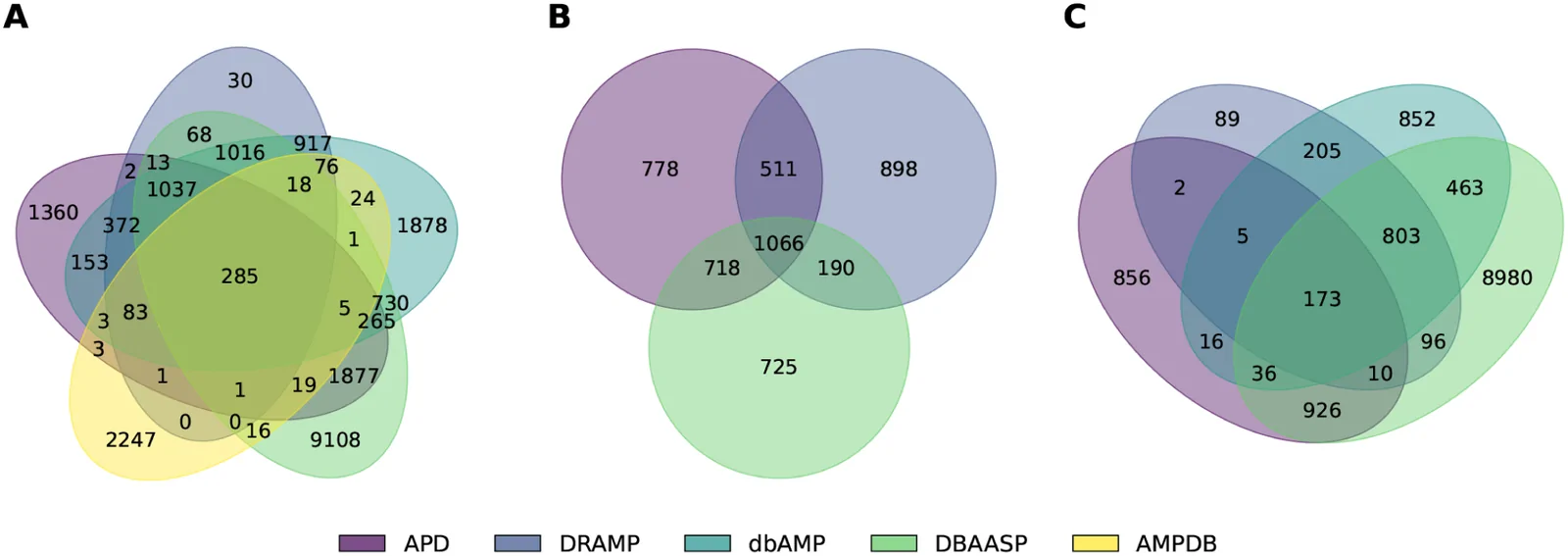
Overlap analysis of ABP sequences across five primary repositories. Each panel shows a Venn diagram illustrating the distribution and overlap of standard antibacterial peptide sequences within APD, DRAMP, dbAMP, DBAASP, and AMPDB, restricted to the aggregated set (A), the natural subpopulation (B), and the synthetic subpopulation (C). Each ellipse represents a database, and each region shows the size of the corresponding intersection. Ellipses are omitted for databases that do not provide the corresponding subpopulation classification. Together, these diagrams reveal the degree of redundancy and uniqueness within the aggregated data set and its natural and synthetic subpopulations, highlighting both the consensus and diversity of curated ABP data across the current database landscape.

From a historical perspective, APD is the earliest database among those evaluated in this study and was one of the first resources to establish systematic criteria for the curation and inclusion of AMPs. Subsequently developed databases introduced their own inclusion and curation criteria, reflecting differences in their scope and objectives. Nevertheless, logically these criteria share several common features, which may partly explain the substantial overlap observed between APD and the other databases. This pattern is particularly evident when natural ABPs are considered. For example, the exclusive pairwise overlap between APD and DBAASP comprises 718 natural sequences, whereas only 190 natural sequences are shared exclusively between DBAASP and DRAMP (Figure 2B). The composition of APD also reflects its stronger emphasis on naturally occurring peptides, which account for approximately 60% of its categorized ABPs. In contrast, DRAMP and DBAASP contain a substantially larger proportion of synthetic peptides, representing approximately 66% and 80% of their categorized ABPs, respectively (Figure 2B–C and Table 1).

DBAASP contained the largest absolute number of database-specific sequences, with 9,108 entries found exclusively in this repository, whereas DRAMP contributed only 30 unique sequences (Figure 2A). Normalization by the total number of ABPs contained in each database further revealed marked differences in database composition. Unique sequences represented only 0.8% of DRAMP, compared with 24.8% of APD and 27.4% of dbAMP. The largest relative proportions of unique sequences were observed for DBAASP and AMPDB, for which database-specific entries represented 63.0% and 80.8% of their respective data sets. These results indicate that both repositories contain substantial sequence content that is not represented in the other major ABP databases examined here. However, the nature of this apparent uniqueness differs between databases. In DBAASP, 8,980 of the 9,108 unique sequences were classified as synthetic, indicating that its high level of exclusivity is largely driven by experimentally designed or modified peptides rather than naturally occurring ABPs. An equivalent distinction could not be established for AMPDB because sequence origin was not systematically annotated as natural or synthetic.

Despite these differences, a core set of 285 sequences was shared across all five databases. Examination of exclusive intersections showed that the largest pairwise overlap occurred between DBAASP and APD, comprising 1,877 sequences. These exclusive intersection values include only sequences shared by the indicated databases and absent from all other repositories, thereby providing a more precise representation of redundancy between individual database combinations. Substantial higher-order intersections were also observed, including 1,016 sequences shared exclusively among DBAASP, dbAMP, and DRAMP, and 1,037 sequences shared exclusively among DBAASP, dbAMP, APD, and DRAMP. Overall, APD and DBAASP were prominent components of the major shared intersections, whereas AMPDB showed comparatively limited participation in the dominant overlapping subsets. This distinct overlap pattern is consistent with differences in the construction and scope of AMPDB, including the incorporation of longer peptide sequences and precursor proteins, which distinguish part of its sequence space from that covered by the other repositories.

To investigate whether these overlaps reflected shared biophysical traits, we performed a comparative analysis of peptide physicochemical properties. The resulting density plots (Figure 3) delineate the distributive characteristics of each database. The specific methodologies for calculating these sequence-derived descriptors are documented in Supplementary file 1.

**Figure 3:**
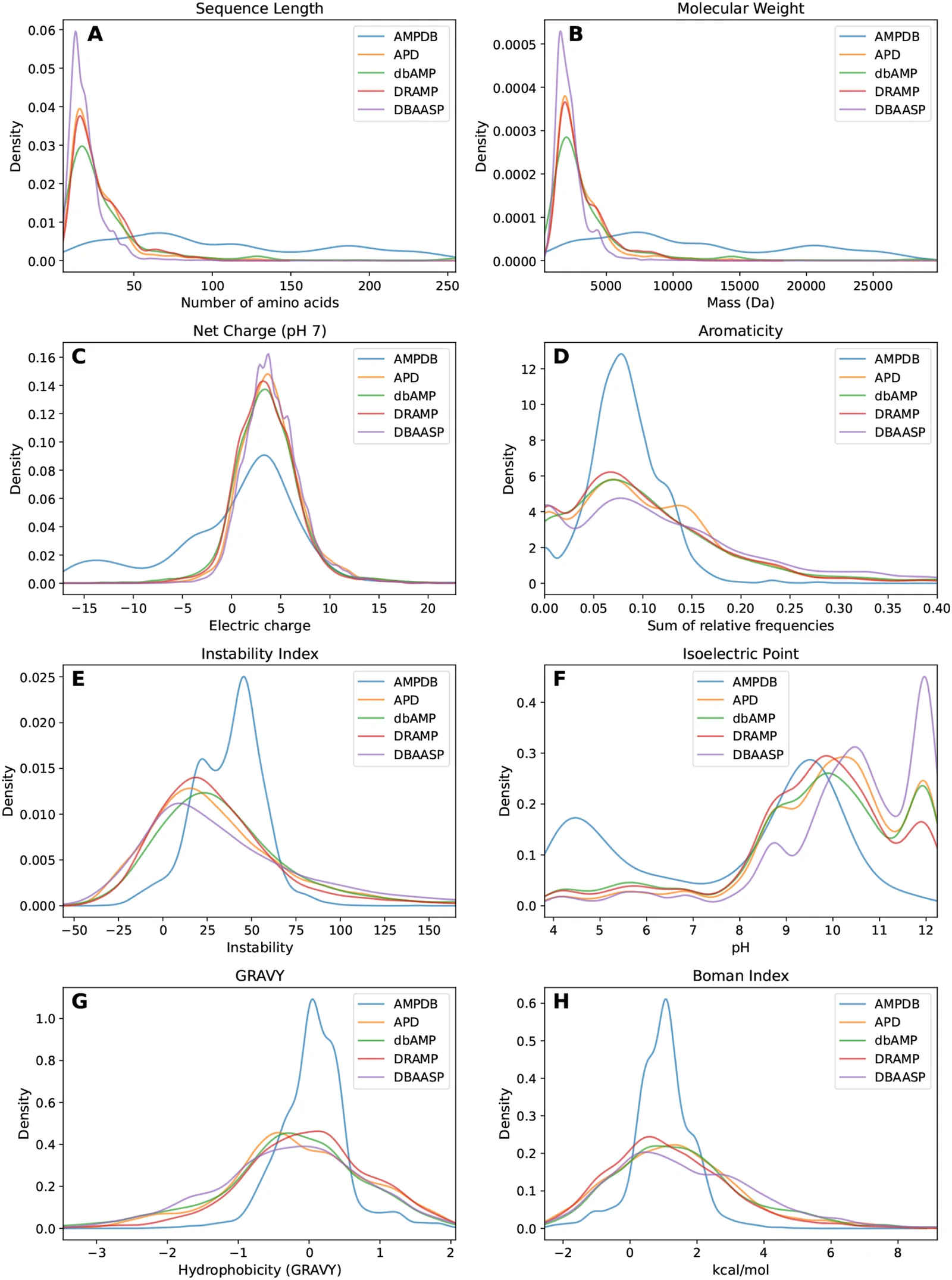
Density distributions of physicochemical attributes across primary ABP repositories. Each panel (A–H) illustrates the probability density function for a specific physicochemical descriptor derived from standard ABP sequences (see Supplementary file 1 for details on how they were computed). The overlaid curves facilitate a comparative assessment of the biophysical landscapes across the five analyzed databases, highlighting regional consensus and divergent property profiles. Variations in these distributions reflect differences in curation priorities and the specialized nature of the curated peptide space within each repository.

The density plots in Figure 3 reveal a consistent distributional overlap among most physicochemical properties across the specialized repositories, with the notable exception of AMPDB, which consistently exhibits divergent patterns. This variance likely stems from its integrative curation model, which draws from general protein databases rather than specific antimicrobial literature. The resulting sequence heterogeneity aligns with the reduced overlaps observed in the Figure 2 intersection analysis. Therefore, AMPDB captures a distinct region of the biophysical landscape, complementing rather than duplicating the other repositories in the aggregated ABP corpus. Sequence length and molecular weight exhibit synchronized distributions across all data sets, confirming the direct linear relationship between these parameters (Figure 3A and 3B). Structural constraints within APD (max 200 residues) and DRAMP (max 100 residues) are clearly visible, while DBAASP is uniquely enriched with very short peptides. In contrast, AMPDB explores the sequence space more broadly, showing no significant preference for specific length clusters. A comprehensive analysis of net charge at pH 7 indicates that, in contrast to the prevalence of cationic ABPs, the AMPDB database exhibits a notably larger subpopulation of negatively charged peptides compared to other databases (Figure 3C). This observation is further corroborated by the appearance of a shoulder on the left side of the isoelectric point plot in AMPDB, which indicates a greater diversity of acidic peptides (Figure 3F).

Analysis of aromaticity and hydrophobicity (GRAVY index) reveals synchronized distributional patterns among the databases, with hydrophobicity curves showing a balanced symmetry around the origin (Figure 3D and 3G). In contrast, AMPDB sequences exhibit a significantly lower variance for these properties despite maintaining similar mean values. This constrained dispersion is also reflected in the instability index and the Boman index, which estimates a peptide’s potential to bind to other proteins (Figure 3E and 3H). Low instability index values across all data sets suggest that the majority of curated ABPs possess moderate predicted structural stability, although AMPDB presents appreciably higher values. Boman index values are centered around a mean of approximately 1 kcal/mol across databases, with a right-skewed distribution indicating that most peptides exhibit moderate or lower potential for peptide–protein interaction, while a smaller subpopulation, represented in the right tail, shows a markedly higher predicted interaction potential. This divergent behavior is likely driven by AMPDB’s distinct amino acid composition (depleted in arginine and enriched in aspartate and glutamate) and its comparatively smaller sample size, both of which are consistent with its particular curation scope, which incorporates longer sequences and precursor proteins.

To investigate the distributional anomalies in AMPDB, we performed a secondary analysis of its unique sequences by correlating length with electrostatic profiles. As shown in Figure 4, sequences with a net charge below −5 exhibit a significantly higher mean length (175 residues) than the remaining sequences (92 residues). Inspection of the names annotated in AMPDB identified a higher prevalence of bactericidal permeability-increasing (BPI) proteins within this long-chain anionic cohort. BPI proteins are a specialized class of membrane-disrupting proteins present in polymorphonuclear leukocyte granules and active against Gram-negative bacteria.^113^ This classification was taken directly from AMPDB and then cross-checked against UniProtKB entries annotated as bactericidal permeability-increasing protein bearing from 5 to 255 residues, where the vast majority of the AMPDB BPI sequences were found with an exact match (e.g., UniProt accession A0A8T2JUT4). Furthermore, the BPI sequences present in AMPDB exhibited no overlap with entries from the other four databases, none of which contained sequences matching these UniProt BPI entries. The presence of these proteins highlights the potential for significant architectural variation within curated antimicrobial data sets. For sequences with a charge higher than −5 (Figure 4), the length distribution reveals a complex multimodal topology, with a global peak located around 70 residues. The distribution exhibited herein underscores the inherent structural heterogeneity of the data set, which is evidenced by the presence of diverse peptide classes exhibiting a wide spectrum of chain lengths. Consequently, the characterization of these subpopulations, as illustrated in Figure 4, is imperative for the development of next-generation predictive tools. This inherent heterogeneity necessitates a transition toward more granular, feature-aware modeling strategies, which can accurately capture the diverse physicochemical signatures found within expanding AMP families.

**Figure 4:**
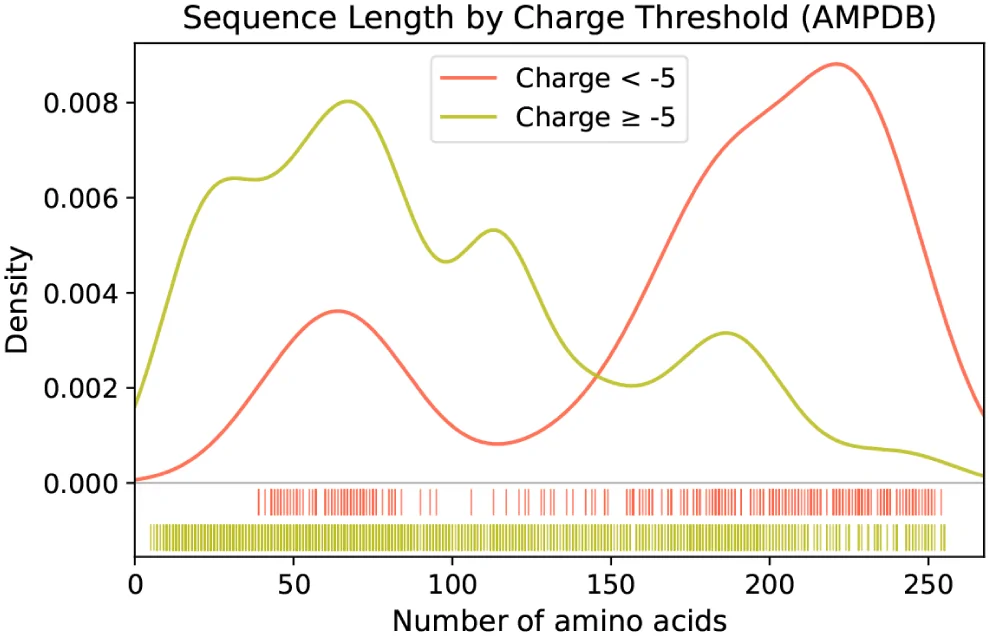
Comparative sequence length density based on a charge threshold from AMPDB. Individual sequence lengths are represented as tick marks in two stacked lanes beneath the density curves, colored to match each subpopulation, showing the exact underlying observations alongside the smoothed distributions. Red hue, subpopulation of highly anionic sequences (charge < −5), exhibiting a shift toward higher sequence lengths with a main peak at 211 residues and a minor one at 64. Green hue, complementary subpopulation of sequences with charge ≥ −5, characterized by discrete peaks at 31, 67, 113, and 186 residues, the tallest being at 67.

## Comparative Analysis of Antibacterial and Nonantimicrobial Peptide Sequences

To investigate the distinctive features that separate ABP sequences from non-AMPs, we compared our consolidated ABP data set against standard non-AMPs extracted from UniProt. The resulting density profiles (Figure 5) reveal pronounced distributive shifts in physicochemical properties between the two classes. A two-sample Kolmogorov–Smirnov test was employed to evaluate the null hypothesis of identical distributions. The results confirmed that ABPs and non-AMPs differ significantly across all analyzed properties (*p* < 10^–283^ in all instances). This comparative framework is critical for characterizing the feature importance and the biophysical decision boundaries utilized by the ML architectures evaluated in the following sections. The observed distributional separation suggests that these physicochemical descriptors carry meaningful discriminative signal, contributing to the capacity of classifiers to distinguish ABPs from non-AMPs.

**Figure 5:**
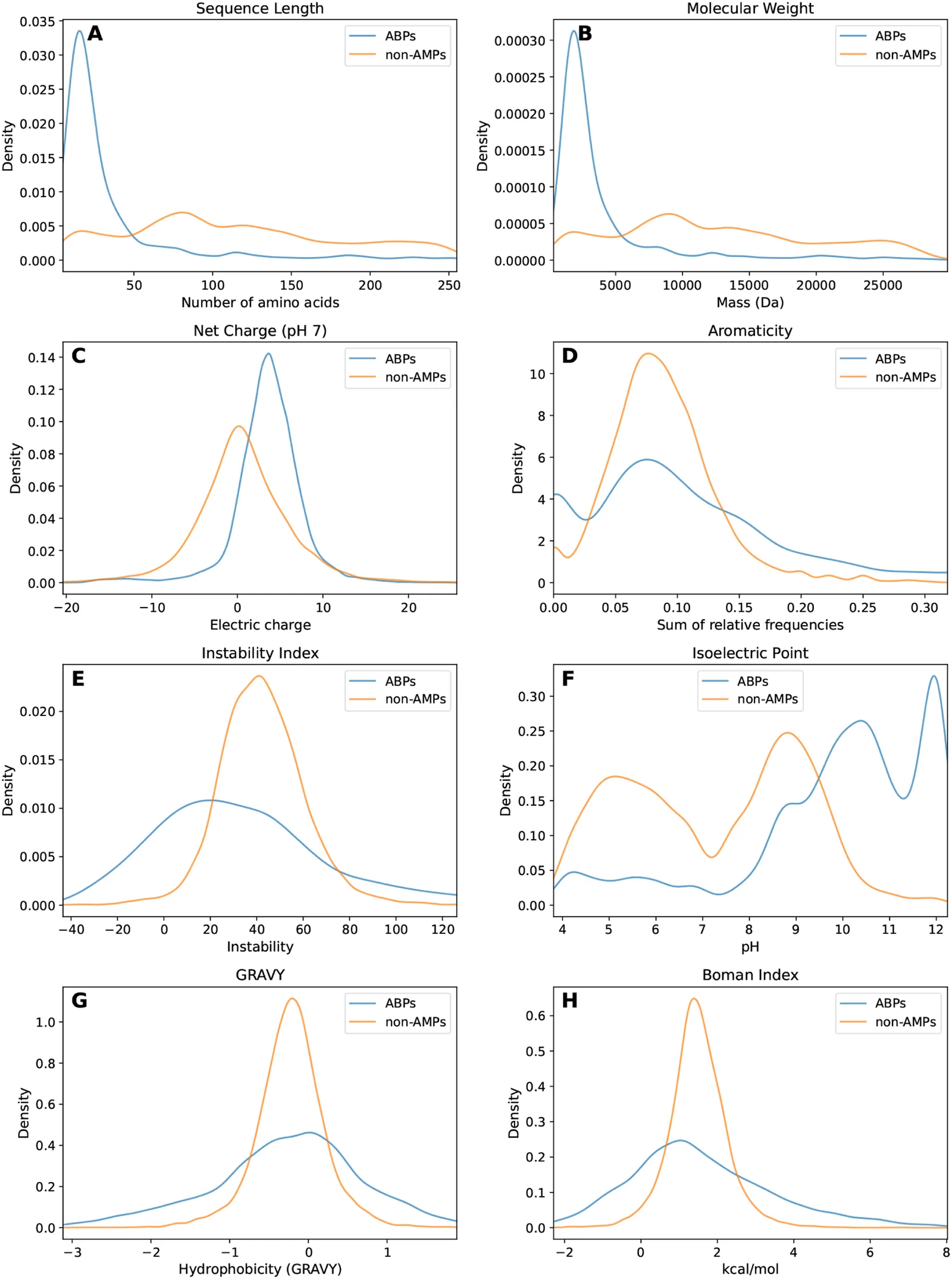
Comparative density distributions of physicochemical attributes for ABPs and nonantimicrobial sequences. Each panel (A–H) illustrates the probability density function for a specific physicochemical descriptor, comparing the integrated ABP data set (derived from five primary repositories) against a curated cohort of non-AMPs from UniProt, comprising 21,608 and 15,184 entries, respectively.

Comparative analysis of the sequence space shows that non-AMPs feature higher molecular weight and broader length distributions than ABPs, which consist mainly of peptides under 50 residues (Figure 5A and 5B). Although net charge profiles at pH 7 overlap, the non-AMP data set displays a measurable shift toward anionic values (Figure 5C). Isoelectric point data further support this trend because the non-AMP cohort remains biased toward the acidic range despite both sets being bimodal (Figure 5F).

Aromaticity values for non-AMPs are concentrated near 0.07, whereas the ABP population is more widely dispersed across the scale (Figure 5D). Similar to patterns in the AMPDB repository, non-AMPs show remarkably lower variance in instability, hydrophobicity, and Boman indices than the diverse ABP group (Figure 5E, 5G, and 5H). The majority of ABPs exhibit moderate structural stability, whereas non-AMPs include a more significant proportion of less stable peptides, with instability index values equal to or greater than 40. The Boman index reveals a broader range of interaction potential among ABPs, with a mean value of 1 kcal/mol, whereas non-AMPs show a slightly higher mean, closer to 2 kcal/mol, although the majority still fall within the moderate-potential range. Furthermore, the GRAVY index indicates that ABPs also span a broader hydrophobicity range, centered closer to zero, a characteristic that supports the formation of amphipathic structures necessary for membrane-active mechanisms.

These features collectively suggest that ABPs exhibit a higher propensity for interaction with biological targets and for exerting activity through diverse mechanisms. Their specific cellular targets likely depend on their individual hydrophobic and electrostatic profiles. At the same time, the observed variability across databases suggests the inclusion of functionally diverse peptides with substantially different physicochemical characteristics.

Significant differences between ABPs and non-AMPs are evident across all analyzed properties, pointing to fundamental distinctions rooted in their biological functions. Therefore, these properties serve as highly informative descriptors for class differentiation based on amino acid sequence features. It is important to note that AMPDB sequences show physicochemical distributions more similar to the non-AMP set. This observation indicates the presence of atypical or functionally distinct peptides and underscores the need for rigorous data set characterization during the development of predictive models.

## Machine Learning and Deep Learning Tools for Predicting AMPs

The computational landscape for AMP research involves a broad variety of frameworks designed for activity prediction, functional classification, and quantitative estimation of potency (Figure 6). These methodologies also extend to the generative design of new sequences. These tools are systematically organized into two main groups according to their modeling techniques. Tools that use classical ML algorithms (i) such as random forest (RF) or support vector machine (SVM) utilize predefined sequence features to capture physicochemical and structural properties. In contrast, modern DL approaches (ii) learn latent representations from raw sequence data or utilize the transformative power of protein language models (PLMs). Many current frameworks adopt hybrid strategies by combining ML and DL components for improved robustness. Additional refinements such as motif scoring and structural constraints are often implemented to guide peptide optimization and ensure biological relevance.

**Figure 6:**
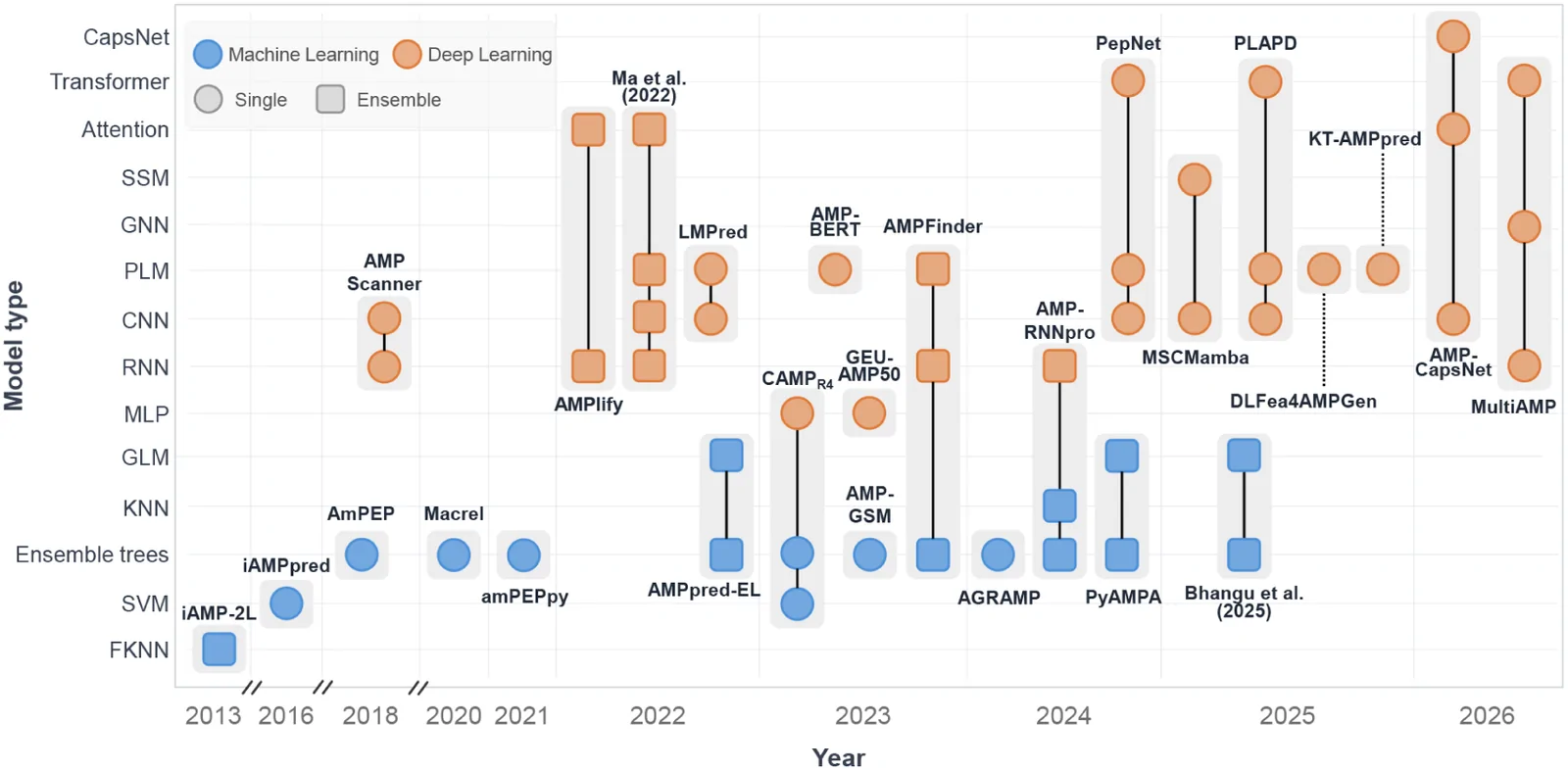
Chronological evolution of computational architectures for AMP prediction. This visualization shows the evolution of predictive models from 2013 to 2026. The *Y*-axis lists the fundamental neural and algorithmic architectures, while the *X*-axis indicates the year of publication. The blue and orange markers distinguish between machine learning and deep learning approaches, respectively. Single-model predictors are represented by circles, while squares indicate ensemble methods. Methods that integrate various architectural components are linked by vertical connectors, and the shaded regions delineate the specific components of each tool. The data reveal a significant technological shift from classical machine learning classifiers to advanced deep learning classifiers. CapsNet, capsule network; SSM, state space model; GNN, graph neural network; PLM, protein language model; CNN, convolutional neural network; RNN, recurrent neural network; MLP, multilayer perceptron; GLM, general linear model; KNN, k-nearest neighbors; SVM, support vector machine; FKNN, fuzzy k-nearest neighbors.

Within the first methodological group (i), early approaches include **AntiBP**,^114^ **iAMP-2L**,^28^ and **iAMPpred**.^29^ AntiBP established a precedent for ABP prediction by employing quantitative matrices, SVMs, and multilayer perceptrons over the N-terminus, C-terminus, or whole sequence of antibacterial peptides from the APD. iAMP-2L implements a two-layer multilabel classifier based on a fuzzy k-nearest neighbors algorithm: the first layer discriminates AMPs from non-AMPs, whereas the second assigns predicted peptides to one or more of ten functional categories. In a different approach, iAMPpred employs SVMs combined with information gain-based feature selection to provide specialized classifiers for antibacterial, antiviral, and antifungal activities.

Several subsequent tools rely on tree ensemble models to enhance predictive robustness. **AmPEP** ^30^ uses a RF classifier with correlation-based feature selection, while **amPEPpy** ^33^ provides a Python version of AmPEP with a refined model training phase. **AMPpred-EL** ^37^ combines four gradient boosting machine classifiers with a logistic regression meta-model in a two-layer ensemble scheme. **AMP-GSM** ^41^ constructs RF models through a feature grouping and scoring strategy to identify informative descriptor sets for predicting antimicrobial and anti-inflammatory peptides.

Other tools extend this paradigm with additional functionalities. The **CAMP**_**R4**_ ^38^ database offers three separate prediction algorithms, namely a RF, a SVM and a multilayer perceptron, distinguishing between natural and synthetic peptides prior to prediction. **Macrel** ^32^ provides an end-to-end pipeline for AMP discovery from genomic and metagenomic data using a RF classifier; it operates on peptide sequences and raw reads or contigs and includes a hemolytic activity classifier. Furthermore, **PyAMPA** ^45^ integrates RF-based modules for motif identification, antimicrobial activity prediction against several bacterial species, hemolytic activity and toxicity prediction, half-life estimation via multivariable regression, and sequence optimization through mutagenesis and evolutionary algorithms. More specialized tools include **AGRAMP**,^43^ designed to identify peptides active against phytopathogenic bacteria and applied to discover putative AMPs encoded in the citrus (*Citrus sinensis*) genome. More recently, the framework proposed by **Bhangu et al. (2025)** ^48^ combines an extreme gradient boosting classifier with motif ranking and LASSO (least absolute shrinkage and selection operator) regression to iteratively generate candidate antimicrobial peptides.

Collectively, the field has undergone a significant evolution from classical ML toward sophisticated molecular engineering. This trajectory began with simple sequence discrimination but has progressed into a paradigm of high algorithmic complexity. The adoption of ensemble methods such as RF and extreme gradient boosting has proved essential to better manage the inherent noise in biochemical data sets. Ultimately, the discipline is shifting from passive discovery toward active design. Contemporary frameworks now utilize evolutionary algorithms and regression to optimize the generation of novel peptides for highly specific targets.

Deep learning strategies in the second group (ii) prioritize the extraction of sequence representations directly from raw amino acid data. **AMP Scanner** ^31^ was among the first models to apply deep neural networks to AMP classification, combining convolutional layers with long short-term memory (LSTM) units. A reduced-alphabet variant based on k-means clustering of amino acids was also introduced to simplify the feature space. Progressively, PLMs pretrained on large protein databases became central components for AMP classification tasks. **LMPred** ^36^ employs embeddings derived from three PLMs, specifically BERT (Bidirectional Encoder Representations from Transformers),^115^ T5,^116^ and XLNet,^117^ each independently combined with a convolutional neural network. **AMP-BERT** ^39^ uses a fine-tuned BERT architecture for AMP classification and enables interpretation of predictions through self-attention analysis. **PLAPD** ^49^ leverages embeddings from the ESM-2 protein language model and integrates subsequent convolutional and transformer modules for feature extraction. **KT-AMPpred** ^51^ separates AMP identification and functional classification using a fine-tuned T5 PLM and transfer learning for predicting antibacterial, antifungal, and antiviral peptides. In contrast, **GEU-AMP50** ^40^ implements a simple multilayer perceptron with two hidden layers designed to classify short peptides of up to 50 amino acids.

Concurrently, various ensemble and multistage architectures emerged. **AMPlify** ^34^ integrates five submodels incorporating bidirectional LSTM layers with multihead and context attention modules, and was applied to AMP discovery in the genome of *Rana [Lithobates] catesbeiana*. The pipeline developed by **Ma et al. (2022)** ^35^ combines convolutional networks with LSTM layers and an attention mechanism together with a BERT model to identify novel AMP candidates within human gut microbiome data. **AMPFinder** ^42^ employs a cascaded architecture in which a RF classifier first identifies AMPs, followed by a DL model predicting ten functional classes using PLM embeddings combined with bidirectional LSTM, gated recurrent unit layers, and an attention mechanism. **AMP-RNNpro** ^44^ uses stacked ensemble learning in which predictions from multiple ML classifiers are used as input to a recurrent neural network meta-classifier. Beyond ensemble strategies, other complex architectures were proposed. **PepNet** ^46^ combines embeddings from a pretrained T5 model with features extracted via a residual dilated convolution block, feeding a transformer module to predict antimicrobial, anti-inflammatory, and toxic peptides. **AMP-CapsNet** ^52^ introduces a capsule network-based architecture with sequence encoding and channel-attention convolutional modules, using amino acid composition or dipeptide composition as input features.

Finally, some recent works extend beyond functional classification. **MSCMamba** ^47^ focuses on minimal inhibitory concentration (MIC, defined as the lowest concentration of an antimicrobial compound required to inhibit visible bacterial growth under in vitro conditions) prediction by combining a multiscale convolutional network with a selective state space model. **DLFea4AMPGen** ^50^ proposes a pipeline for *de novo* peptide generation with potential antibacterial, antifungal, and antioxidant properties, based on a BERT-like PLM combined with explainability approaches that are specific from ML. **MultiAMP** ^53^ integrates ESM-2 embeddings with bidirectional LSTM-based sequence modeling and structural features from geometric graph neural networks using cross-attention and transformer modules to jointly predict antimicrobial activity and secondary structure. It also enables rational peptide design and has been applied to identify novel AMPs from ocean-derived proteins.

A comprehensive list of the tools assessed, together with associated metadata, is provided in Supplementary file 2.

Taken together, these tools illustrate the rapid methodological evolution and diversity of computational approaches for AMP modeling. Although many frameworks report high performance in their modeling tasks, the training corpora used, the variety of AMP activities targeted, and the model evaluation protocols vary substantially. Therefore, they are not directly comparable, making it difficult to assess them in a rigorous and fair manner. In addition, functional subtypes within AMPs differ in their physicochemical and biological properties, meaning that predicting each activity effectively constitutes a distinct classification problem. This heterogeneity underscores the importance of systematic benchmarking under standardized conditions and of focusing on a specific AMP class to reduce the intrinsic variability of the underlying data sets.

## A Standard Evaluation Framework for AMP Predicting Tools

For a rigorous and fair evaluation of predictive tools, each method should meet two essential requirements: (i) the data sets used for model development should be publicly available, with training, validation, and test partitions clearly defined; and (ii) the trained prediction model should be directly accessible and usable by independent evaluators. Evaluated tools that failed to meet either of these criteria were flagged accordingly. This approach was strictly followed to mitigate bias and maintain transparency in the interpretation of all evaluation outcomes.

Regarding the first requirement, knowing the exact set of instances used to train each model is essential to construct a rigorous evaluation set that excludes all training examples. Furthermore, the partitioning of the data set into classes must be clearly specified, ensuring that incompatible classes (e.g., AMPs vs non-AMPs, or training vs test sets) do not overlap. Ideally, curated data sets should be deposited in a public repository and linked to the study through a persistent URL or DOI, ensuring long-term availability and consistency with the data described in the publication. Among the 26 tools assessed, only 16 provided their training data sets directly, whereas 13 provided test data sets; and just two, PepNet and MultiAMP, linked their data sets via a DOI. In practice, even nominally accessible data required substantial retrieval effort. The URL reported for AmPEP data sets was inaccessible and the data had to be retrieved through the GitHub repository linked in the Macrel documentation, which reuses those data sets. For PepNet, the linked repository contained several distinct, unclearly labeled corpora, and manual inspection was required to identify which partitions were actually used for training and evaluation. Similarly, the data set provided by Ma et al. (2022) was not explicitly identified as a training or test partition. In the case of iAMP-2L, the data set was embedded in Supplementary file 2 and had to be extracted manually.

Beyond accessibility, an exhaustive analysis of data leakage, encompassing overlaps between positive and negative classes within a functional category and between training and test partitions, was conducted across all 17 tools for which data were available. Among these, 8 presented overlap between positive and negative classes, with the most affected cases reaching globally overlapping fractions of 22.0%, 3.2%, and 1.9%; and 5 presented overlap between training and test partitions, with the largest cases reaching 21.8%, 1.5%, and 0.4%. Notably, the tool with the highest class overlap also exhibited the most severe train/test leakage. Complete information on data availability and retrieval for each tool, along with the downloaded data sets, automated download scripts, and data leakage analysis code, is hosted in the project repository. More broadly, the paucity of unique identifiers for peptides underscores the absence of a standardized evaluation framework in this scientific domain, a circumstance that starkly contrasts with the prevailing norm in protein databases.

Regarding the second requirement, the accessibility of predictive models varied considerably across tools, and practical limitations were widespread. Some projects offer web servers that enable rapid prediction, but these often restrict the number of input sequences per submission, as seen with Macrel and AGRAMP, and do not support automation or integration into a standardized benchmarking pipeline. In some cases, such as AGRAMP, results cannot be downloaded and must be manually copied for further analysis. Similar constraints affect tools distributed as local graphical user interface applications, which typically enforce rigid workflows and do not allow isolation of the classification process, as is the case with PyAMPA. Compounding these issues, several web servers referenced in the original publications are no longer accessible, including iAMP-2L, iAMPpred, and AmPEP, while others remain available but with nonfunctional prediction capabilities, as occurs with AMP-RNNpro. Among the 26 tools evaluated, only 15 provide access to source code, and just nine include trained model files. The absence of trained models is a major obstacle to reproducibility. Even when training protocols and original data sets are available, successful reproduction may be hindered by hardware constraints, software dependency issues, or implementation flaws such as logical errors, missing components, or insufficient documentation.

To address these limitations, we developed individualized Docker environments for each tool, respecting the reported dependency requirements while fixing bugs and standardizing the handling of inputs, outputs, and hardware. All preprocessing and prediction steps were integrated into an easy-to-run Nextflow pipeline. Throughout this process, the original logic of each tool was preserved, and published model files were used where available. For the four tools lacking pretrained models, LMPred, AMP-BERT, PLAPD, and KT-AMPpred, separate training pipelines were also developed using the training parameters specified in the original scripts. Ultimately, of the 15 tools providing source code, only AMP-CapsNet could not be tested: the published code covers only the network architecture definition, omitting all data loading and feature extraction steps as well as any trained model file, making the tool impossible to reproduce or run. The remaining 14 tools were successfully integrated into the Nextflow pipeline. AGRAMP and CAMP_R4_, which could not be incorporated into the pipeline due to the absence of local execution environments, were evaluated separately using their prediction servers. Full details on code adaptations, Docker images, and workflow execution are available in the project repository https://github.com/Maximo-7/amp_review.

A specialized evaluation data set was extracted from the complete data set by combining standard ABP sequences from the five primary databases (Table 1) with non-AMP entries from UniProt. Potential data leakage was eliminated by the strict exclusion of any instances identified within the training sets of the evaluated tools. The resulting balanced data set of ABPs and non-AMPs, hereafter referred to as the *evaluation data set*, provided the foundation for our standardized prediction pipeline. This allowed for the extraction of class assignments and probabilities used to annotate the entire data set. By prioritizing the exclusion of overlapping sequences, this framework establishes a robust and unbiased environment for benchmarking the predictive accuracy of diverse AMP predictive tools.

Analyses of predicted probabilities and classification labels from the evaluation data set were conducted to assess performance and identify commonalities among the predictive tools (Figure 7). This process enabled the identification of significant sequence subpopulations, specifically distinguishing between those that were consistently easy to classify and those that posed persistent challenges for the models. By isolating these subsets, the study delineates the current boundaries of the antimicrobial sequence space that remain difficult to model. Such insights are essential for the targeted improvement of future DL and ML frameworks. Thus, while some classifiers like DLFea4AMPGen exhibit an AUROC as low as 24.7%, others such as Macrel achieve a more robust 89.9% (Figure 7A). These findings suggest that current modeling approaches have yet to reach a level of universal reliability. Furthermore, the observation that many published models perform near random levels during independent testing points to a critical disconnect between self-reported accuracy and rigorous validation. Such results reinforce the importance of adopting transparent and standardized evaluation protocols to accurately assess progress in antimicrobial peptide discovery.

**Figure 7:**
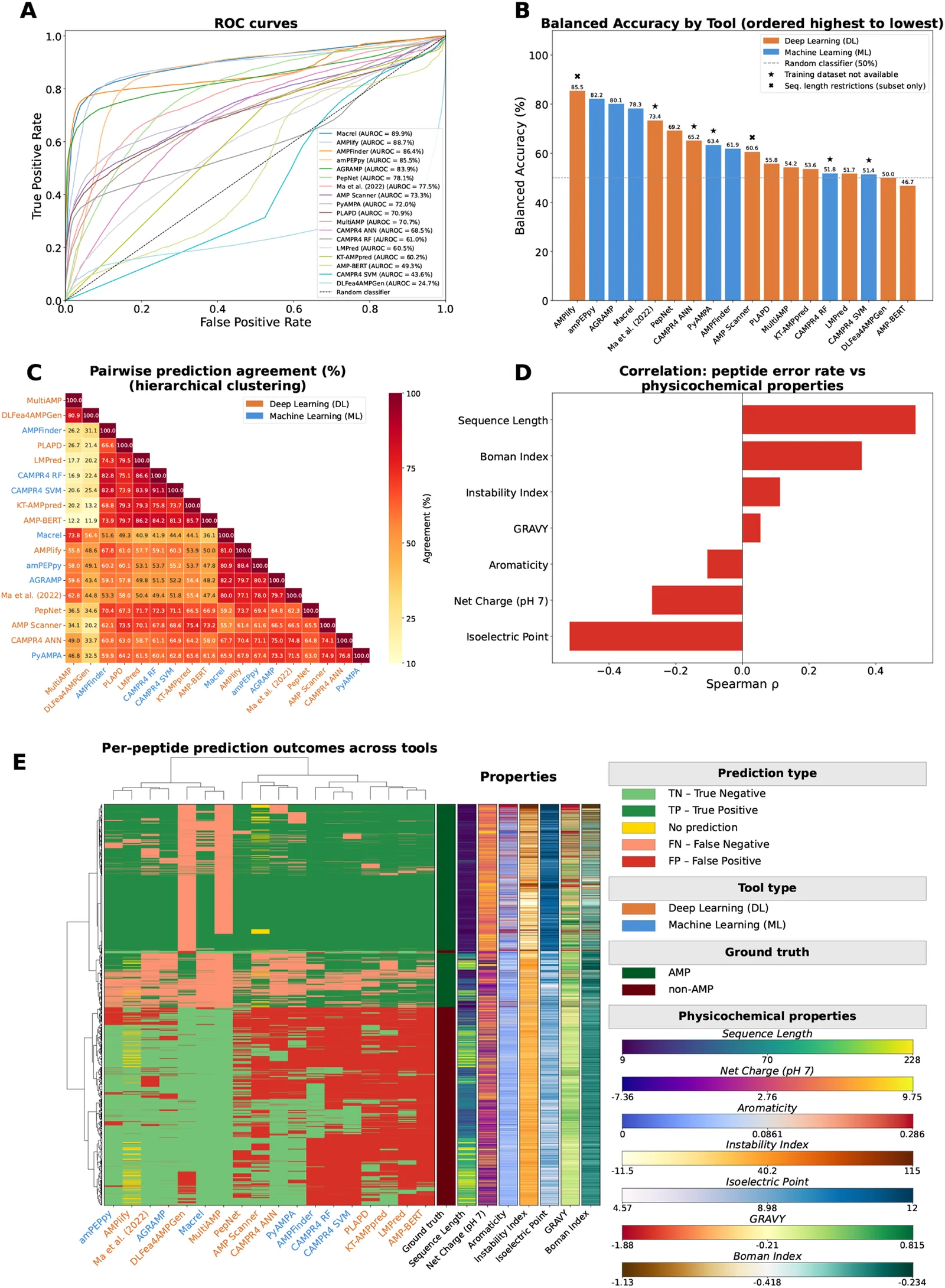
Comprehensive performance and behavioral analysis of 16 antimicrobial peptide (AMP) prediction tools. (A) ROC curves for all 18 evaluated models ranked by AUROC with a dashed diagonal representing the random classifier baseline. (B) Balanced accuracy for each model ordered from highest to lowest. Orange and blue bars distinguish deep learning (DL) and classical machine learning (ML) paradigms, respectively. The star symbol (★) denotes tools with nonpublic training data sets while the cross symbol (**X**) identifies tools evaluated on restricted sequence-length subsets. (C) Pairwise prediction agreement matrix organized via hierarchical clustering using average linkage and a distance metric of 100 minus the agreement percentage. Tool labels are color-coded by methodological class. (D) Spearman correlation analysis between the per-peptide error rate and specific physicochemical properties. Red bars signify statistically significant correlations where *p* < 0.05. (E) Global heatmap of per-peptide prediction outcomes with rows and columns organized by Ward linkage hierarchical clustering. Cell colors indicate true positives (dark green), true negatives (light green), false positives (red), and false negatives (salmon). Yellow cells represent instances where tool-specific length restrictions precluded a prediction. Lateral side strips denote the ground-truth class alongside physicochemical property values clipped to the fifth and 95th percentile range. Specific model configurations were selected for tools offering multiple predictive architectures. For CAMP_R4_, the ANN, RF, and SVM models were evaluated as separate entities. PyAMPA was represented by the AMPValidate model, while the length/count-balanced configuration was utilized for amPEPpy. The LMPred evaluation employed the T5 UniRef50-based model, and AMPFinder was assessed using the stage 1 classifier. For AGRAMP, the 3-g 9-letter model was selected. The ABP-MPB configuration was used for DLFea4AMPGen, whereas the AMP Fine-tuned Model was chosen for KT-AMPpred. Finally, MultiAMP was evaluated using the sequence-only model. These selections ensure that the most representative or highest-performing available versions of each framework are reflected in the benchmark results.

Figure 7B corroborates this picture while enabling a further comparison by methodological class. In terms of balanced accuracy, although the top-ranked model is DL-based, DL does not emerge as a clearly superior paradigm: the three immediately following positions are occupied by classical ML models, and the ranking as a whole shows no consistent methodological trend. This ambiguity is noteworthy given that DL is the predominant approach in the field, with 11 DL models compared to only seven ML models among those reviewed.

Tools evaluated only on restricted sequence-length subsets occupied ranks 1 and 10 in terms of balanced accuracy, whereas tools for which potential overlap between the training data and the evaluation set could not be explicitly excluded were distributed across ranks 5, 7, 8, 14, and 16. The performance of tools in both categories should be interpreted with caution. For tools with uncertain training data, the evaluation set may include sequences used during model development, introducing a risk of data leakage. For tools assessed on restricted length subsets, the reduced evaluation space may underrepresent difficult-to-classify sequences. In both cases, the reported performance may therefore be inflated relative to that expected under a fully independent and unrestricted benchmark.

Preprocessing methodologies vary significantly across the evaluated frameworks (Supplementary file 2). Deep learning models typically prioritize direct numerical encodings (e.g., one-hot or integer encoding) or PLM embeddings to capture positional and inter-residue relationships via attention mechanisms. In contrast, classical machine learning models frequently employ reduced alphabets based on physicochemical similarity or indirect descriptors to represent the sequence. These distinct strategies suggest that primary linear sequences alone may lack the information density required for high-precision prediction. This potential shortfall highlights either a constraint in current algorithmic feature extraction or a necessity for integrating auxiliary structural and functional annotations. Consequently, future predictive architectures should likely incorporate multimodal descriptors to improve the classification of complex antimicrobial sequences.

Pairwise agreement across predictive tools is generally low, averaging 59.7% with a peak of 91.1% between the CAMP_R4_ RF and SVM variants (Figure 7C). Hierarchical clustering reveals a primary group of high-performing tools, including AMPlify, amPEPpy, AGRAMP, and Macrel, which maintain robust intertool agreement above 80%, alongside a significant secondary cluster comprising CAMP_R4_ RF, CAMP_R4_ SVM, KT-AMPpred, and AMP-BERT. In addition, MultiAMP and DLFea4AMPGen form an isolated, highly correlated cluster (80.9%) that nonetheless diverges sharply from all other methodologies. Notably, certain tools demonstrate consistently low agreement with the rest of the cohort. Most strikingly, DLFea4AMPGen records a mean agreement of only 34.7% and appears largely decoupled from the high-performing clusters, showing its highest similarity (56.4%) with Macrel. These findings indicate that while disparate architectures can converge on high accuracy, other models may form clusters due to shared representational strategies or similar training sets. Such patterns suggest that intertool agreement is a product of both biological signal and methodological commonalities within the sequence space.

Physicochemical properties significantly influence predictive error rates as quantified by the Spearman correlations in Figure 7D. Sequence length shows the highest positive correlation with failure (ρ ≈ 0.5), while isoelectric point and net charge exhibit the strongest negative correlations. These results demonstrate that models are most accurate when evaluating short, highly basic peptides but fail progressively as sequences become longer or less cationic. Additionally, increased failure rates are associated with higher Boman and instability indices, as well as reduced aromaticity. This systematic bias underscores a foundational limitation in current training methodologies, which prioritize canonical ABP profiles at the expense of more diverse sequences. To enhance the generalization capabilities of future ML and DL frameworks, training data sets must be expanded to include more uniform distributions across these critical physicochemical parameters, particularly addressing the high error rates associated with noncationic and high-molecular-weight peptides.

The performance patterns of the evaluated tools are summarized in the global error heatmap shown in Figure 7E, where hierarchical clustering reveals a structured geometric distribution of predictive performance. By dividing the heatmap into six functional sectors based on the intersection of tool profiles and peptide categories, the systematic nature of the limitations of current models becomes evident. The left side of the heatmap is dominated by high-performance tools such as AMPlify, Macrel, amPEPpy, AGRAMP, and the framework by Ma et al. (2022). Within this area, the upper-left sector represents a consensus zone where these models accurately identify canonical ABPs. Below this, in the lower-left corner, a region of maximum specificity is defined. In this sector, the leading tools successfully filter out non-AMPs and demonstrate superior ability to distinguish true negatives even among sequences that mimic cationic properties. However, these same models, despite their overall success, consistently fail on a specific subset of atypical ABPs located in the center-left sector, highlighting a critical scientific frontier. These systematic failures suggest that even state-of-the-art architectures reach an information ceiling when encountering peptides that deviate from classical physicochemical archetypes.

Conversely, the right-hand side encompasses the majority of the evaluated instruments, which are distinguished by substantial performance biases. The upper-right quadrant demonstrates a false consensus, wherein the tools accurately classify numerous ABPs, primarily due to an excessively permissive classification threshold. Notable exceptions to this phenomenon include DLFea4AMPGen and MultiAMP, which exhibit an extremely conservative bias, resulting in the presence of visible vertical bands of false negatives. Moreover, the lower-right quadrant signifies a domain replete with false positives, as over half of the reviewed instruments are incapable of accurately identifying sequences that are not AMPs.

Finally, the center-right sector indicates a blind spot, where tools with a false-positive bias fail to identify the same difficult ABPs as top-tier models. This finding indicates that a nonselective model does not exhibit enhanced biological sensitivity. Rather, it has been demonstrated that the model is capable of recognizing canonical patterns while remaining blind to atypical antimicrobial sequences.

## Conclusions and Recommendations

This review has presented a comparative analysis of AMP databases, with a specific focus on ABPs, followed by the application of a standardized framework to evaluate the ability of existing AMP prediction tools to distinguish ABP sequences from non-AMP sequences. Based on our analysis, several conclusions can be drawn, together with specific recommendations to improve both AMP databases and AMP prediction tools.

First, regarding AMP databases, only five out of the 13 resources evaluated met the inclusion criteria of accessibility and active maintenance (Supplementary file 1). This finding highlights the need for sustained funding, curation, and technical support to preserve these resources for the scientific community. Among the five databases retained for analysis, we observed that almost all sequences from DRAMP were also present in other databases. In contrast, the remaining four databases contained a considerable number of unique sequences that were not represented elsewhere. These results indicate that, despite the existence of several AMP repositories, there is still a clear need for a comprehensive ABP-focused database that integrates sequences currently dispersed across different resources. It is also relevant to acknowledge that database redundancy may arise from multiple sources, including repeated experimental reports, identical peptide sequences identified in different organisms, and inconsistencies or errors in database curation. Therefore, database identifiers should not be assumed to represent unique amino acid sequences, and sequence-level deduplication should be performed before integrating data sets or developing and evaluating machine learning models. Importantly, identical sequences with different experimental annotations should be carefully assessed to preserve relevant biological information.

Furthermore, and perhaps more importantly, AMP databases contain valuable information beyond peptide sequences, including MIC values, structural annotations, toxicity data, target organisms, and experimental validation details. However, this information remains underused for AMP prediction and model development. One major limitation is that most databases primarily allow sequence downloads, while offering limited or no access to structured metadata through APIs, standardized formats, or programmatic query systems. To fully exploit the biological and experimental information contained in these resources, future AMP databases’ updates should provide interoperable access to curated metadata, including standardized end points, downloadable annotation tables, and clear documentation. Such improvements would facilitate the development of more informative prediction tools, enable better benchmarking, and support the design of models that move beyond simple AMP/non-AMP classification toward predictions of antibacterial activity, potency, toxicity, and therapeutic potential.

These differences in database composition are not merely descriptive but may directly affect AI-based AMP discovery. Training sets dominated by synthetic peptides can constrain the learned sequence space toward design-associated features, potentially reducing the ability of models to recognize the diversity of naturally occurring ABPs. Recent observations that genome-predicted AMPs can display amino acid signatures closer to synthetic than to natural peptides illustrate how biases in the underlying databases may propagate into downstream predictions. Thus, careful characterization of natural and synthetic sequences is essential to mitigate the ″garbage in, garbage out″ problem in AMP machine learning.^118^

Several efforts have been made to design and implement new computational tools for AMP detection. However, our analysis reveals a clear discrepancy between the performance reported for many tools in their original publications and their performance under an independent, standardized evaluation framework. This gap underscores the need for external benchmarking based on transparent data sets, consistent evaluation metrics, and independent test sets that are strictly excluded from model training.

Beyond predictive performance, our review also highlights a broader problem of reproducibility and model availability in the field. Only 16 of the 26 tools reviewed were suitable for evaluation, and even within this subset, some tools lacked essential information, such as clearly reported training data sets or ready-to-use models. These limitations make it difficult to determine whether benchmark sequences were previously seen during training, to reproduce the original results, and to compare methods under fair and consistent conditions. Improving the transparency, accessibility, and documentation of AMP prediction tools should therefore be considered a priority for future methodological development.

Therefore, we encourage researchers developing AMP prediction tools to evaluate their models using our benchmark data set, while ensuring that these sequences, or highly similar homologues, are excluded from their training data. Such practices would reduce the risk of data leakage, improve the reliability of reported performance estimates, and facilitate fairer comparisons among prediction tools.

Taken together, these results lead to several fundamental conclusions. First, there remains considerable room for improvement in the ability of current models to specifically distinguish ABPs from non-AMPs. This is particularly evident when models are challenged by nonantimicrobial sequences with similar composition or physicochemical properties. This lack of specificity results in the saturation of false positives observed in the right-hand sectors of the global error landscape. Furthermore, the identification of the atypical ABPs located in the center-left sector of the heatmap represents a critical scientific frontier (Figure 7E). Because even top-performing models fail systematically in this region, it is clear that current architectures have reached an information ceiling regarding these sequences. Second, further progress in the field will require more carefully designed training corpora that ensure balanced and representative coverage across the full range of relevant peptide properties, especially sequence length, charge, hydrophobicity, and amino acid composition. Such data sets should also minimize redundancy and avoid overlap between training and evaluation sets, as these factors can lead to inflated estimates of predictive performance.

Finally, our results suggest that features derived solely from the linear amino acid sequence may be insufficient to fully capture the biological determinants of antimicrobial activity. Incorporating secondary and tertiary structural information could provide a richer and more biologically meaningful basis for classification. More broadly, these findings indicate that sequence representation may be as critical as the choice of predictive algorithm itself.

In this context, emerging protein language models and structure-aware approaches represent promising directions for future AMP prediction. Their potential impact, however, will depend on the availability of data sets and the ability of researchers to integrate information regarding sequence, structure, and function as a whole.

## Supplementary Material





## Data Availability

Full details on the code adaptations, Docker images, and workflow execution are provided in the project repository: https://github.com/Maximo-7/amp_review. The data generated in this study, referred to above as the ″complete data set″ and the ″evaluation data set″, are available in the GitHub repository and have also been deposited in Zenodo with an associated DOI 10.5281/zenodo.20966530.

## Notes

Criteria for including antimicrobial peptide databases, the list of included and excluded databases, methodologies for calculating the sequence-derived physicochemical descriptors and density plots, and the filter used for the UniProt query, excluding antimicrobial peptides from the search (PDF)
Comprehensive list of the tools assessed, together with associated metadata in tabular format (XLSX)
