# Supplementary material for: Antimicrobial Peptide Databases and Prediction Tools: Toward a Standard Evaluation Framework
Source: J Chem Inf Model. 2026 Oct 27;66(21):13708–26. doi: 10.1021/acs.jcim.6c02141 (PMC13753515; doi:10.1021/acs.jcim.6c02141)
Supplement: Supplementary file 1 [file ci6c02141_si_001.pdf]

Supporting Information: Antimicrobial peptide databases and prediction tools: Toward a standard evaluation framework

Alejandro Cisterna-García*[2], Álvaro M. González-López*[1], Aspasia Vozi[2,7,8], María A. Esteban[6], Adrian Egli[3], Catherine Jutzeler[2], Jose Palma[1], Álvaro Sánchez-Ferrer[4], and Juan A. Botía†[1,5]

[1]Departamento de Ingeniería de la Información y las Comunicaciones, Universidad de Murcia, 30100, Murcia, Spain
[2]Biomedical Data Science Lab, Department of Health Sciences and Technology (D-HEST), ETH Zurich, 8092, Zurich, Switzerland
[3]Institute of Medical Microbiology, University of Zurich, 8006, Zurich, Switzerland
[4]Departamento de Bioquímica y Biología Molecular-A, Facultad de Biología, Universidad de Murcia, 30100 Murcia, Spain
[5]Department of Neurodegenerative Disease, UCL Institute of Neurology, London WC1E 6BT, UK
[6]Departamento de Biología Celular e Histología, Facultad de Biología, Universidad de Murcia, 30100, Murcia, Spain
[7]Department of Intensive Care and Neonatology, Children's Research Center, University Children's Hospital Zurich, University of Zurich, 8008, Zurich, Switzerland
[8]SIB Swiss Institute of Bioinformatics, 1015, Lausanne, Switzerland

*These authors contributed equally to this work
†Email: juanbot@um.es

Brief description of supplementary material

- `supplementary_file_1.pdf`: Criteria for including amtimicrobial peptide databases, the list of included and excluded databases, methodologies for calculating the sequence-derived physicochemical descriptors and density plots, and the filter used for the UniProt query, excluding antimicrobial peptides from the search (PDF).
- `supplementary_file_2.xlsx`: Comprehensive list of the tools assessed, together with associated metadata in tabular format (XLSX).

Criteria to include antimicrobial databases

Criteria: Ability to filter for antibacterial peptides, ongoing updates and maintenance at least every 3 years and accessibility. Eligibility was assessed in early 2026.

Databases meeting the selection criteria (Pass)

- APD [1] – last update: January 2026
- DBAASP [2] – last update: 2026
- dbAMP [3] – last update: June 2024
- DRAMP [4] – last update: July 2025
- AMPDB [5] – last update: April 2023

Databases not meeting the selection criteria (Not Pass)

- CAMP [6] – no updates since 2022; limited accessibility and reliance on predicted data
- PeptideAtlas [7] – inability to filter and download specifically antibacterial peptides
- LAMP [8] – inaccessible; last update in 2013
- ADAM [9] – data download not accessible
- YADAMP [10] – inaccessible, last update uncertain
- ANTIMIC [11] – inaccessible, last update uncertain
- SATPdb [12] – data download unavailable; last update uncertain (~2015)
- Bactibase [13] – last update in 2017

Methodologies for calculating the sequence-derived descriptors

The physicochemical properties analyzed were inferred directly from the amino acid sequences and are listed below. All properties, with the exception of sequence length and the Boman index, were computed using the `ProteinAnalysis` class from the `Bio.SeqUtils.ProtParam` module of the Biopython library [14]. Boman index was calculated using the `peptides.py` implementation, which follows the Boman formulation based on amino-acid solubility values derived from Radzicka and Wolfenden [15, 16].

- **Sequence length**: the total number of amino acid residues in the sequence.
- **Molecular weight**: the sum of the molecular masses of all residues in the peptide.
- **Net charge (pH 7)**: the overall electric charge of the peptide at pH 7.

- **Aromaticity**: a measure of the relative abundance of aromatic residues, defined according to Lobry and Gautier (1994) [17], as the sum of the relative frequencies of phenylalanine (Phe), tyrosine (Tyr), and tryptophan (Trp) in the sequence.
- **Instability index**: an estimate of protein stability as defined by Guruprasad et al. (1990) [18]; values above 40 indicate an unstable protein and are associated with a short half-life.
- **Isoelectric point**: the pH at which the peptide has a net charge of zero.
- **GRAVY (Grand Average of Hydropathy)**: a measure of the overall hydrophobic or hydrophilic character of the peptide, calculated according to Kyte and Doolittle (1982) [19], as the arithmetic mean of the hydropathy values of all residues in the sequence.
- **Boman index**: an estimate of the peptide's potential to bind to other proteins or membranes, computed by averaging the solubility values for all residues in a sequence.

Density plots were generated using the `pandas.DataFrame.plot.density` method [20, 21], which produces a Kernel Density Estimate (KDE) plot using Gaussian kernels with automatic bandwidth determination. KDE is a non-parametric statistical method that uses kernel smoothing to estimate the probability density function of a random variable. Although sequence length is inherently a discrete variable, KDE was also applied to its distribution, treating it as continuous for visualization purposes, as this produced a smoother and more readable plot than a histogram.

Criteria to select sequences from UniProt

The following UniProt query was used to search for non-AMP sequences:

```
length:[5 TO 255] NOT (keyword:KW-0929) NOT (keyword:KW-0211) NOT (keyword:KW-0044)
AND (keyword:KW-0964) NOT (keyword:KW-0930) NOT (keyword:KW-0295) NOT (keyword:KW-0878)
NOT (keyword:KW-0078) NOT (keyword:KW-0081) NOT (keyword:KW-0425)
```

This query included sequences with a length between 5 and 255 residues and annotated with the keyword Secreted (KW-0964), while excluding sequences annotated with any of the following keywords:

- Antimicrobial (KW-0929)
- Defensin (KW-0211)
- Antibiotic (KW-0044)
- Antiviral protein (KW-0930)
- Fungicide (KW-0295)
- Amphibian defense peptide (KW-0878)
- Bacteriocin (KW-0078)
- Bacteriolytic enzyme (KW-0081)
- Lantibiotic (KW-0425)

References

(1) Wang, G.; Schmidt, C.; Li, X.; Wang, Z. APD6: the antimicrobial peptide database is expanded to promote research and development by deploying an unprecedented information pipeline. *Nucleic Acids Research* **2026**, *54*, D363–D374, DOI: 10.1093/nar/gkaf860.

(2) Pirtskhalava, M.; Amstrong, A. A.; Grigolava, M.; Chubinidze, M.; Alimbarashvili, E.; Vishnepolsky, B.; Gabrielian, A.; Rosenthal, A.; Hurt, D. E.; Tartakovsky, M. DBAASP v3: database of antimicrobial/cytotoxic activity and structure of peptides as a resource for development of new therapeutics. *Nucleic Acids Research* **2020**, *49*, D288–D297, DOI: 10.1093/nar/gkaa991.

(3) Yao, L.; Guan, J.; Xie, P.; Chung, C.-R.; Zhao, Z.; Dong, D.; Guo, Y.; Zhang, W.; Deng, J.; Pang, Y.; Liu, Y.; Peng, Y.; Horng, J.-T.; Chiang, Y.-C.; Lee, T.-Y. dbAMP 3.0: updated resource of antimicrobial activity and structural annotation of peptides in the post-pandemic era. *Nucleic Acids Research* **2025**, *53*, D364–D376, DOI: 10.1093/nar/gkae1019.

(4) Ma, T.; Liu, Y.; Yu, B.; Sun, X.; Yao, H.; Hao, C.; Li, J.; Nawaz, M.; Jiang, X.; Lao, X.; Zheng, H. DRAMP 4.0: an open-access data repository dedicated to the clinical translation of antimicrobial peptides. *Nucleic Acids Research* **2025**, *53*, D403–D410, DOI: 10.1093/nar/gkae1046.

(5) Mondal, R. K.; Sen, D.; Arya, A.; Samanta, S. K. Developing anti-microbial peptide database version 1 to provide comprehensive and exhaustive resource of manually curated AMPs. *Scientific Reports* **2023**, *13*, 17843, DOI: 10.1038/s41598-023-45016-3.

(6) Gawde, U.; Chakraborty, S.; Waghu, F. H.; Barai, R. S.; Khanderkar, A.; Indraguru, R.; Shirsat, T.; Idicula-Thomas, S. CAMPR4: a database of natural and synthetic antimicrobial peptides. *Nucleic Acids Research* **2023**, *51*, D377–D383, DOI: 10.1093/nar/gkac933.

(7) Desiere, F.; Deutsch, E. W.; King, N. L.; Nesvizhskii, A. I.; Mallick, P.; Eng, J.; Chen, S.; Eddes, J.; Loevenich, S. N.; Aebersold, R. The PeptideAtlas project. *Nucleic Acids Research* **2006**, *34*, D655–D658, DOI: 10.1093/nar/gkj040.

(8) Zhao, X.; Wu, H.; Lu, H.; Li, G.; Huang, Q. LAMP: A Database Linking Antimicrobial Peptides. *PLoS ONE* **2013**, *8*, e66557, DOI: 10.1371/journal.pone.0066557.

(9) Lee, H.-T.; Lee, C.-C.; Yang, J.-R.; Lai, J. Z. C.; Chang, K. Y. A Large-Scale Structural Classification of Antimicrobial Peptides. *BioMed Research International* **2015**, *2015*, 475062, DOI: 10.1155/2015/475062.

(10) Piotto, S. P.; Sessa, L.; Concilio, S.; Iannelli, P. YADAMP: yet another database of antimicrobial peptides. *International Journal of Antimicrobial Agents* **2012**, *39*, 346–351, DOI: 10.1016/j.ijantimicag.2011.12.003.

(11) Brahmachary, M.; Krishnan, S. P. T.; Koh, J. L. Y.; Khan, A. M.; Seah, S. H.; Tan, T. W.; Brusic, V.; Bajic, V. B. ANTIMIC: a database of antimicrobial sequences. *Nucleic Acids Research* **2004**, *32*, D586–D589, DOI: 10.1093/nar/gkh032.

(12) Singh, S.; Chaudhary, K.; Dhanda, S. K.; Bhalla, S.; Usmani, S. S.; Gautam, A.; Tuknait, A.; Agrawal, P.; Mathur, D.; Raghava, G. P. SATPdb: a database of structurally annotated therapeutic peptides. *Nucleic Acids Research* **2016**, *44*, D1119–D1126, DOI: 10.1093/nar/gkv1114.

(13) Hammami, R.; Zouhir, A.; Le Lay, C.; Ben Hamida, J.; Fliss, I. BACTIBASE second release: a database and tool platform for bacteriocin characterization. *BMC Microbiology* **2010**, *10*, 22, DOI: 10.1186/1471-2180-10-22.

(14) Cock, P. J. A.; Antao, T.; Chang, J. T.; Chapman, B. A.; Cox, C. J.; Dalke, A.; Friedberg, I.; Hamelryck, T.; Kauff, F.; Wilczynski, B.; de Hoon, M. J. L. Biopython: freely available Python tools for computational molecular biology and bioinformatics. *Bioinformatics* **2009**, *25*, 1422–1423, DOI: 10.1093/bioinformatics/btp163.

(15) Boman, H. G. Antibacterial peptides: basic facts and emerging concepts. *Journal of Internal Medicine* **2003**, *254*, 197–215, DOI: 10.1046/j.1365-2796.2003.01228.x.

(16) Radzicka, A.; Wolfenden, R. Comparing the polarities of the amino acids: side-chain distribution coefficients between the vapor phase, cyclohexane, 1-octanol, and neutral aqueous solution. *Biochemistry* **1988**, *27*, 1664–1670, DOI: 10.1021/bi00405a042.

(17) Lobry, J. R.; Gautier, C. Hydrophobicity, expressivity and aromaticity are the major trends of amino-acid usage in 999 Escherichia coli chromosome-encoded genes. *Nucleic Acids Research* **1994**, *22*, 3174–3180, DOI: 10.1093/nar/22.15.3174.

(18) Guruprasad, K.; Reddy, B. V.; Pandit, M. W. Correlation between stability of a protein and its dipeptide composition: a novel approach for predicting in vivo stability of a protein from its primary sequence. *Protein Engineering* **1990**, *4*, 155–161, DOI: 10.1093/protein/4.2.155.

(19) Kyte, J.; Doolittle, R. F. A simple method for displaying the hydropathic character of a protein. *Journal of Molecular Biology* **1982**, *157*, 105–132, DOI: 10.1016/0022-2836(82)90515-0.

(20) Pandas development team, T. pandas-dev/pandas: Pandas, version latest, 2020, DOI: 10.5281/zenodo.3509134.

(21) McKinney, W. In *Proceedings of the 9th Python in Science Conference*, ed. by van der Walt, S.; Millman, J., 2010, pp 56–61, DOI: 10.25080/Majora-92bf1922-00a.
